# The Role of Sodium Oxybate in Idiopathic Hypersomnia: A Case Report Showing Improvement of Excessive Daytime Sleepiness and Reduced Symptoms

**DOI:** 10.7759/cureus.45976

**Published:** 2023-09-26

**Authors:** Michael Vera Ricaurte, Jamshaid Akhtar, Palak Patel, Akshay Sundaram, Kirti K Kharel, Mohammad Kagzi

**Affiliations:** 1 General Medicine, Universidad Central de Venezuela, Caracas, VEN; 2 Internal Medicine, Midwest Sleep and Wellness Clinic, Chicago, USA; 3 Internal Medicine, Sir Sayajirao General (SSG) Hospital, Vadodara, IND; 4 Internal Medicine, Kasturba Medical College, Manipal, IND; 5 Internal Medicine, Nobel Medical College Teaching Hospital, Biratnagar, NPL; 6 Sleep Medicine, Advocate Condell Medical Center, Chicago, USA

**Keywords:** idiopathic hypersomnia, sodium oxybate, modafinil, multiple sleep latency test, epworth sleepiness scale

## Abstract

Our aim is to report the clinical profile and outcome of patients diagnosed with idiopathic hypersomnia (IH). Idiopathic hypersomnolence is a complex, debilitating, and uncommon sleep disorder manifested mainly by chronic excessive daytime sleepiness (EDS).

This paper reports on the treatment of a patient with idiopathic hypersomnia who was treated with low sodium oxybate (LXB) due to a lack of response to the first-line drug modafinil. This patient, who presented with worsening excessive daytime sleepiness, sleep drunkenness, and sleep disturbances, was diagnosed with idiopathic hypersomnia by overnight polysomnography (PSG) and a multiple sleep latency test (MSLT). Stimulant agent modafinil was prescribed along with sleep hygiene education. Her symptoms did not respond to modafinil, and she was switched to a recently approved newer medication, i.e., low sodium oxybate.

## Introduction

Idiopathic hypersomnia (IH) is a type of primary central hypersomnolence disorder (CHD) that can manifest in different ways. The main symptom is excessive daytime sleepiness (EDS), and patients may experience prolonged nighttime sleep, unrefreshing naps lasting longer than one hour, sleep inertia, and cognitive impairment. These additional features can vary among individuals and contribute to the heterogeneity of the disorder [1].

Although IH shares some similarities with other CHDs such as narcolepsy types 1 and 2, it is important to focus on its distinct clinical characteristics. Unlike narcolepsy, IH is not typically associated with cataplexy, hypnagogic or hypnopompic hallucinations, sleep paralysis, or disrupted night sleep [2]. Instead, patients with IH often experience unrestful sleep and sleep inertia, which is characterized by a feeling of grogginess and impaired performance upon awakening after naps [3]. Polysomnography (PSG) and especially multiple sleep latency test (MSLT) play a crucial role in differentiating IH from narcolepsy and other potential causes of EDS, such as obstructive sleep apnea (OSA) [4]. By focusing on the unique clinical features of IH, healthcare providers can accurately diagnose and treat this debilitating disorder.

The pathogenesis of IH as it happens in other CHD is not clear. Consequently, the drug treatment up to now has mainly focused on reducing the more debilitating symptoms such as EDS with stimulant medications such as modafinil [5]. However, the first idiopathic hypersomnia-specific treatment, lower sodium oxybate (LXB), was recently approved by the United States (US) Food and Drug Administration (FDA) [6], which marks a significant step toward more research for the treatment of this disorder. Sodium oxybate is the sodium salt of gamma hydroxybutyrate, which is an endogenous compound and metabolite of the neurotransmitter gamma-aminobutyric acid (GABA). It is hypothesized that its therapeutic effects on cataplexy and excessive daytime sleepiness are mediated through GABA-B actions at noradrenergic and dopaminergic neurons, as well as at thalamocortical neurons.

Due to the paucity of data and the limited understanding of IH, case reports can provide valuable insights into the clinical presentation, diagnosis, and management of this condition. We present a case of a 27-year-old female patient with IH focusing on the different treatment approaches, especially in her response to lower sodium oxybate, aiming to contribute to the growing knowledge of IH and to promote a better understanding of this condition.

## Case presentation

A 27-year-old female was referred to our clinic for a sleep consult with complaints of difficulty initiating sleep, excessive daytime sleepiness, and sleep drunkenness for 10 years. These symptoms were insidious in onset, initially mild in severity, and later progressed over time. Excessive daytime sleepiness was associated with tiredness and exhaustion throughout the day and unrefreshing naps. Lately, symptoms have worsened in a way that affects her routine life while watching television, talking on the phone, conversing with somebody, and waiting at a stop while driving.

She has neither experienced loud snoring nor witnessed gasping for air and wheezing during her sleep. She does not report sudden weakness in her arms, legs, and neck. She does not report feeling anything different while falling asleep or waking up from sleep. Her Epworth Sleepiness Scale (ESS) score was 19 at her initial visit. She used to take cannabidiol (CBD) gummies to enhance her sleep quality, although it was not beneficial. She also reported that she used to take melatonin at night to sleep better but could not tolerate it as she was feeling paralyzed in the middle of the night.

Her past medical history includes hypothyroidism, which is well controlled with tablet levothyroxine (37.5 micrograms) as per her endocrinologist, and her recent thyroid-stimulating hormone (TSH) is within normal limits. She has also been diagnosed with psoriasis and migraine headaches, which have been well controlled with medications. Her past medical history also revealed irritable bowel syndrome (IBS) and lactose intolerance, for which she was advised to take a gluten- and lactose-free diet along with probiotics. She occasionally consumes alcohol about twice a week but does not consume it to fall asleep.

Physical examination findings did not reveal any remarkable findings such as sinus tenderness, increased neck circumference, elevated body mass index (BMI), or any other findings that may explain the abovementioned symptoms.

A home sleep study was performed (Table 1) to determine the etiology of the patient’s complaints of daytime sleepiness. The results of the home sleep study revealed an Apnea-Hypopnea Index (AHI) of 3.5 and a Respiratory Disturbance Index (RDI) of 3.5 that was inconclusive for obstructive sleep apnea (OSA) as the cause for her symptoms. The patient was scheduled for a full-night polysomnogram (PSG) and a multiple sleep latency test (MSLT) for further evaluation of her symptoms.

**Table 1:** Home sleep study results REI: Respiratory Event Index, AHI: Apnea-Hypopnea Index, RERA: respiratory effort-related arousal, CAI: Central Apnea Index, RDI: Respiratory Disturbance Index, TRT: total recording time, TIB: time in bed, MT: monitoring time

Time and durations			
Lights off clock time	10:05:05 PM	TRT	576 minutes
Lights on clock time	7:41:05 AM	TIB	576 minutes
		MT	576 minutes

Summary							
AHI	3.5	RDI=AHI+RERA	3.5	CAI	0.5	Lowest Desat	81
REI is the number of respiratory events per hour. OAl is the number of obstructive apneas per hour. CAl is the number of central apneas per hour. Lowest Desat is the lowest blood oxygen level that lasts at least two seconds.							

Respiratory events									
	Index (#/hour)	Total # of events	Mean duration (seconds)	Maximum duration (seconds)	# of events by position				
					Supine	Prone	Left	Right	Up
Central apneas	0.5	5	13.6	15.5	4	/	0	1	0
Obstructive apneas	0.7	7	90.1	556.5	5	/	0	2	0
Mixed apneas	0.2	2	13	15	2	/	0	0	0
Hypopneas	2.1	20	22.4	56.5	20	/	0	0	0
Apneas+hypopneas	3.5	34	34.5	556.5	31	/	0	3	0
RERAs	0	0	0	0	0	/	0	0	0
Total	3.5	34	34.5	556.5	31	/	0	3	0
Time in position (minutes)					376.1	/	32.6	166.3	1
REI in position					4	/	0	1	0

Until then, the patient was counseled to practice proper sleep hygiene measures and to return to our clinic in six weeks if there was no improvement in symptoms, and she was started on 100 mg of modafinil for her daytime symptoms. The patient came to us three weeks before her follow-up still reporting no improvement of her symptoms despite taking modafinil and practicing proper sleep hygiene practices. Her dosage of modafinil was adjusted to 200 mg, and six weeks later, she came back to us for her routine follow-up still reporting no improvement in her daytime sleepiness. We did not increase the dose of modafinil because she was suffering side effects associated with increased doses of modafinil such as headache and nausea. Her Epworth Sleepiness Scale score was still 19.

At that time, an overnight polysomnogram (Table 2) was performed that showed a Respiratory Disturbance Index of 11.3, which was inconclusive for obstructive sleep apnea. We then performed an MSLT (Table 3) on the following day. This MSLT consisted of three daytime nap opportunities spaced at two-hour intervals beginning at 6:06:34 AM. The following was observed.

**Table 2:** Overnight polysomnography results REM: rapid eye movement, RDI: Respiratory Disturbance Index, CA: central apnea, OA: obstructive apnea, MA: mixed apnea, REI: Respiratory Event Index, AHI: Apnea-Hypopnea Index, RERA: respiratory effort-related arousal, CAI: Central Apnea Index, NREM: non-rapid eye movement, WASO: wake after sleep onset, TST: total sleep time, EEG: electroencephalogram

Sleep architecture baseline portion of the study				
Total sleep time	229 minutes	Sleep efficiency	59.50%	-
Total time in bed	384.7 minutes	Latency to sleep onset	151.5 minutes	-
WASO	4.2 minutes	Latency to REM onset	214.5 minutes	-
Sleep stages	Minutes	% TST	% Normal	-
N1	5	2.2	5	-
N2	160	69.9	50	-
N3 (stage 3 and 4)	20	8.7	5-15	-
REM sleep	44	19.2	20-25	-
Arousals	Number	# per hour		-
EEG arousals (total)	158	41.4		-
Respiratory (A+H+RERAs)	8	2.1		-
Snore arousals	0	0		-
Leg arousals	0	0		-
Non-specific arousals	150	39.3		-
Respiratory events	# per hour	-	-	-
Apneas	1	-	-	-
Hypopneas	9	-	-	-
Total apneas/hypopneas	10	-	-	-
Apnea-Hypopnea Index	2.6	-	-	-
RERA index	8.6	-	-	-
RDI-AHI+RERA	11.3	-	-	-

**Table 3:** MSLT results MSLT: multiple sleep latency test, REM: rapid eye movement

Nap data			
Lights out (hh:mm:ss)	Sleep onset (hh:mm:ss)	Sleep latency (minutes)	REM latency (minutes)
6:06:34 AM	6:10:04 AM	3.5	/
7:31:13 AM	7:32:43 AM	1.5	/
9:05:06 AM	9:05:12 AM	0.1	/
Latency data			
Mean sleep latency (3 values)	1.70 minutes	-	-
Average REM latency (0 values)	/ minutes	-	-

Individual latencies for the naps were as follows: nap #1, 3.5 minutes; nap #2, 1.5 minutes, and nap #3, 0.1 minutes. MSLT revealed a mean sleep latency of 1.70 minutes with no REM onsets observed. The patient remained awake in between naps well during the study.

After confirming the diagnosis through these studies and seeing no improvement with a 200 mg dose of modafinil, the treatment was switched to low sodium oxybate (Xywav) to be taken before bedtime. Three weeks later, the patient reported an improvement in excessive daytime sleepiness (EDS), stating that their naps were more restful, and reported improvement in sleep drunkenness and cognitive problems. It is important to note, however, that the patient experienced adverse effects including headaches and morning nausea.

## Discussion

Idiopathic hypersomnia (IH) is a chronic neurological disorder that manifests as pathologic excessive daytime sleepiness with or without prolonged night sleep durations. Diagnosis involves thorough clinical evaluation to exclude other differential diagnoses, in addition to objective sleep testing. Current International Classification of Sleep Disorders-Third Edition (ICSD-3) diagnostic criteria require the presence of EDS for more than three months, absence of cataplexy, and confirmatory objective sleep testing [7]. Our patient experienced 10 years with EDS and an Epworth Sleepiness Scale (ESS) score of 19, which classifies her within the range of severe EDS. She also denied having suffered cataplexy episodes.

Confirmatory objective sleep testing includes the multiple sleep latency test (MSLT) and/or polysomnography (PSG). Performing nocturnal polysomnography is crucial to rule out more common causes of excessive daytime sleepiness (EDS), such as obstructive sleep apnea. Additionally, it can reveal characteristic features of idiopathic hypersomnia (IH), such as a total sleep time exceeding 660 minutes over a 24-hour period with a short sleep latency [7,8]. In our case, a home sleep study (Table 1) and an overnight PSG (Table 3) revealed an Apnea-Hypopnea Index of 3.5 and a Respiratory Disturbance Index of 3.5, which was inconclusive for other causes of EDS, such as obstructive sleep apnea.

The multiple sleep latency test (MSLT) also plays a crucial role in diagnosing idiopathic hypersomnia, as it can reveal characteristic findings such as a mean sleep latency of <8 minutes and <2 short sleep onset REM periods (SOREMP), which refers to REM sleep period occurring ≤15 minutes after the onset of sleep. These findings contrast and help differentiate IH from narcolepsy, where the mean sleep latency is similar but presents more than two SOREMPs [7,9]. An MSLT test was performed on the patient being studied (Table 2), with a preceding overnight sleep PSG (Table 3) revealing a mean sleep latency of 1.70 minutes with no REM onsets observed (no SOREMPs). These results confirm the diagnosis of IH according to the ICSD-3 guidelines [7] and help us rule out narcolepsy as a differential diagnosis.

Treatment options for IH include both non-pharmacological and pharmacological measures. Non-pharmacological interventions include avoiding even slight sleep deprivation, keeping a light/dark cycle, maintaining a regular nocturnal sleep schedule, general self-care, and sleep hygiene. However, up to now, these measures have not shown scientific evidence of benefit [10]. Regardless, a study survey conducted in 2017 found that 96% of participants with self-reported IH used non-pharmacological strategies, including caffeine (82%), daytime naps (81%), scheduled nighttime sleep (75%), exercise (58%), temperature control (47%), and diet (40%) [11].

Pharmacological interventions include mainly modafinil and low sodium oxybate. One of the first published investigations on the treatment of IH was a small non-standardized study of modafinil in 1988, and it was not until 2009 that randomized clinical trials (RCTs) began on this topic [12]. Although modafinil is approved in the United States for the treatment of EDS associated with narcolepsy or obstructive sleep apnea [13] and is the only treatment for IH recommended by the 2021 American Academy of Sleep Medicine (AASM) guidelines [14], its evidence is based on a few small RCTs [15,16]. The largest (N = 71) RCTs about the efficacy of modafinil in IH was published in 2021 and demonstrated an improvement in IH symptoms, including significantly increased sleep latency on the MSLT, decreased Epworth Sleepiness Scale (ESS) scores, and decreased daytime naps, compared with placebo [16].

On the other hand, low sodium oxybate is the only drug approved by the FDA specifically for the treatment of IH [6]. The efficacy of this drug is based on a larger study published in 2022. A phase 3, multicenter, placebo-controlled, double-blind, randomized withdrawal clinical trial (N = 154) evaluated the effect of this drug on IH symptoms. The primary effectiveness endpoint was the improvement in ESS score at the end of the trial, and as the secondary effectiveness endpoint, better outcomes on daytime functioning and work-activity impairment were observed [17].

The patient in this case report was advised to practice proper sleep hygiene measures but showed no improvement in symptoms when she returned to the clinic after six weeks. Initially, she was prescribed modafinil at a dose of 100 mg, which was later increased to 200 mg. However, there was still no improvement in her symptoms, as indicated by the unchanged ESS score of 19 during that consultation. Since the 200 mg dose of modafinil did not yield any positive results, the treatment was switched to low sodium oxybate (Xywav) to be taken before bedtime. After three weeks, the patient reported an improvement in excessive daytime sleepiness (EDS), sleep drunkenness, and cognitive impairment. Her ESS score improved to 14 after switching to low sodium oxybate. It is worth noting, however, that the patient experienced adverse effects, including headaches and morning nausea.

## Conclusions

IH is a chronic neurological disorder that has a significant impact on the patient’s quality of life. Clinicians often encounter numerous obstacles when it comes to diagnosing and treating this disorder. These challenges range from costly confirmatory tests to the difficulty of developing a treatment plan that is not yet universally established. The inclusion of low sodium oxybates in the treatment options for idiopathic hypersomnia is a significant advancement. However, it is important to conduct larger randomized clinical trials to gain a more conclusive understanding of the effectiveness and side effects of low sodium oxybates (LXB) and stimulants such as modafinil, specifically in the context of idiopathic hypersomnia (IH). Further advancements, especially in establishing consistent criteria for determining treatment success in IH, could also greatly assist future clinical research efforts.
